# Temporal dynamics in psychological assessments: a novel dataset with scales and response times

**DOI:** 10.1038/s41597-024-03888-8

**Published:** 2024-09-27

**Authors:** Zhao Su, Rongxun Liu, Yange Wei, Ran Zhang, Xiao Xu, Yang Wang, Yue Zhu, Lifei Wang, Lijuan Liang, Fei Wang, Xizhe Zhang

**Affiliations:** 1 Early Intervention Unit, Department of Psychiatry, The Affiliated Brain Hospital of Nanjing Medical University, Nanjing, China; 2 School of Biomedical Engineering and Informatics, Nanjing Medical University, Nanjing, China; 3 School of Psychology, Xinxiang Medical University, Xinxiang, Henan China; 4 Department of Psychiatry, The Second Affiliated Hospital of Xinxiang Medical University, Henan Mental Hospital, Xinxiang, China; 5 Laboratory of Psychology, The First Affiliated Hospital of Hainan Medical University, Haikou, Hainan China

**Keywords:** Health care, Risk factors

## Abstract

The growing prevalence of mental health issues underscores the need for innovative screening methods. Large-scale, internet-based psychological screening has emerged as a vital tool to accurately determine morbidity rates and facilitate early diagnosis of mental disorders. However, conventional psychological screening methods often struggle with non-genuine responses and lack objective metrics. To bridge this gap, we have compiled a novel dataset derived from an expansive screening initiative at Xinxiang Medical University. The study, conducted from February 27 to March 17, 2021, yielded a dataset comprising responses from 24,292 students to four well-established psychological scales—PHQ-9, GAD-7, ISI, and PSS. A distinctive feature of this dataset is the inclusion of response time data, which captures the temporal dynamics of participants’ interactions with the scales, offering valuable insights into their response behaviour. The release of this dataset offers a substantial opportunity for researchers in the domains of psychology and public health to explore new insights into mental health, scale reliability, and the dynamics of psychological assessment.

## Background & Summary

In recent years, the incidence of psychological disorders has been on the rise, prompting widespread societal concern^1^. Concurrently, with advancements in technology and the ubiquity of the internet, online psychological assessments have emerged as a practical tool for preliminary screenings of mental health status in the general population^2^. These online evaluations not only offer individuals a convenient and swift avenue to understand their psychological well-being, but they also effectively cover a broader demographic. Additionally, early detection and diagnosis through such platforms can lead to timely interventions and treatments, potentially mitigating the progression of the disorder and its impact on an individual’s quality of life^3^. Thus, leveraging online psychological assessments as an early screening mechanism holds significant promise in addressing the escalating challenges of mental health disorders.

While large-scale psychological screenings are essential, the assessment scales they utilize inherently pose challenges. Due to influences such as the environment, internet-based assessment systems still exhibit a certain gap in their results compared to clinical diagnoses^4^. For instance, studies have shown that for screening purposes, using internet-based assessment systems can yield reliable diagnostic outcomes, but often at the expense of sensitivity^5^. During large-scale screenings, where questionnaires are answered in a free environment, a certain percentage of individuals might not answer the questions earnestly. The proportion of careless response (CR) varies widely due to numerous factors, ranging from 1% to 50%^6–10^. A meta-analysis study on alcohol indicated that 11.7% of the sample comprised CRs^11^. Such a rate of CRs can also significantly impact the research outcomes. A particular study revealed that partialling out CRs significantly changed the observed relationships among substantive variables^12^. Maniaci and Rogge found a notable increase in the power of regression after removing samples with CRs^13^. Therefore, identifying and handling CR is a crucial step before analysis.

Response time is a frequently used metric in cognitive psychology experiments, represents the duration required for a participant to react to a stimulus or complete a given task^14^. In the realm of cognitive psychology, response time often serves as a dependent variable, influenced by other independent variables like stimulus exposure duration^15^. Numerous datasets from such psychological experiments are publicly accessible^16,17^. In contemporary internet-based scale screening systems, it’s feasible to collect the time participants take to answer each query, thus deriving a response time sequence as a form of objective behavioural data. In a 2019 Kaggle competition centred around a survey in the data science domain, the total response time participants took to complete the questionnaire was documented as a pivotal variable^18^. This data affords researchers a fresh perspective, enabling exploration from a novel, more objective dimension than solely the options chosen by participants. Without this additional layer of data, the subtle intricacies between individuals might remain elusive. Research has indeed delved into the interplay between response time and participants’ selections^19^.

By recording response times when participants answer scales, this data can be utilized to identify CRs^20^. Furthermore, since self-assessment scales often face issues with participant subjectivity bias, response times can serve as an indicator of a participant’s level of certainty regarding an answer. To delve deeper, since traditional mental health measurement methods based on aggregate or dimensional scores tend to neglect heterogeneity^21^, recent years have witnessed the advent of many computerized adaptive testing scale forms rooted in Item Response Theory (IRT)^22^. Response times, as objective data, can also supplement such computerized adaptive testing. In our prior studies, we employed response time data to architect machine learning models predicting Insomnia Index (ISI) scale outcomes, achieving an accuracy of 0.743^23^. We observed a conspicuous absence of large-scale datasets meticulously recording response times for each question in the field of psychology. This gap poses a hindrance to the in-depth exploration of datasets related to extensive psychological screenings.

In 2021, a detailed psychological screening was conducted at Xinxiang Medical University, encompassing over 20,000 participants. This study involved the collection of data using four established scales - the Patient Health Questionnaire-9 (PHQ-9), the General Anxiety Disorder-7 (GAD-7), the Insomnia Severity Index (ISI), and the Perceived Stress Scale (PSS) - while meticulously recording the response times for each question administered to the subjects. This dataset serves several pivotal roles: 1. Investigate the mental health status of Chinese university students during the COVID-19 pandemic; 2. Identifying invalid responses, which can be gleaned from irregularities in response times^24–27^; 3. Classifying subtypes based on patterns of answering behaviour; 4. Informing the redesign of scales based on response patterns, thereby enhancing the efficiency and accuracy of the questionnaires. This endeavour bridges traditional assessment methodologies with modern data-driven approaches, promising a more nuanced understanding of psychological health metrics.

## Methods

In the pursuit of collecting a large-sample dataset, this study was meticulously designed to gauge the emotional states of university students across four distinct dimensions: depression, anxiety, sleep quality, and stress. Additionally, response times to survey questions were recorded to provide an objective measure of behavioural data. The data collection process spanned 3 weeks, ensuring a comprehensive capture of the targeted metrics.

### Data collection

The data was collected from a psychological screening project organized by Xinxiang Medical University, which was conducted across the entire campus. Notifications were disseminated to students in each class via their counselors. Participation in the project was voluntary for the students. This study has been approved by the Ethics Committee of Xinxiang Medical College of Henan Province (XYLL-2020235). Informed consent was obtained online from all participants prior to survey administration, including agreement for data sharing and dissemination of their data for scientific research. We ensured adherence to all necessary ethical considerations and informed consent procedures throughout the study.

The dataset targeted the university’s enrolled undergraduate and graduate students over approximately three weeks. Due to the constraints imposed by the COVID-19 pandemic, all data collection activities were limited to campus premises. Students were given the flexibility to choose the timing and location for completing the questionnaires. At the conclusion of the data collection phase, a total of 24,367 participants had successfully completed the survey.

### Collection settings

#### Data collection apparatus

A web-based application was developed for the purpose of this study. Participants were able to access the survey via a QR code or a URL in their web browser. This application served as the medium for both administering the questionnaires and collecting the ensuing data. Upon entering the application, participants first completed the informed consent form. Following this, they completed the survey in the following order: demographic questions - > PHQ-9 - > GAD-7 - > PSS - > ISI.

#### Measurement instruments

The psychological scales employed in this study included the Patient Health Questionnaire-9 (PHQ-9)^28,29^, the General Anxiety Disorder-7 (GAD-7)^30,31^, the Perceived Stress Scale (PSS)^32,33^ and the Insomnia Severity Index (ISI)^34,35^. We used the Chinese versions of these scales. Detailed descriptions are shown in Supplement Table. The complete content of the scales, including the text of the questions, response options, and scoring details, can be found in the GitHub repository.

#### Data collection setting

Students were granted the autonomy to select the time and place of their choosing to complete the survey, which allowed for a free and unstructured data collection environment.

#### Data generation process

During the survey, the web application diligently recorded each participant’s response to every item. This was accompanied by the precise measurement of the response time for each question. The methodology involved logging a timestamp upon the participant’s arrival at a new question, followed by a subsequent timestamp upon selection of a response. The interval between these two timestamps, denoted in seconds and precise to two decimal places, constituted the response time for the respective question.

### Data quality control

Participants were required to complete all demographic information and fully respond to all four scales. Out of the total 24,367 collected responses, 75 participants were excluded due to missing values. Consequently, the final dataset includes 24,292 participants. It was acknowledged that network conditions could potentially influence response time recordings, however, given that data collection was conducted within the confines of a single campus, regional disparities were considered negligible, thereby minimizing the impact of network variability on the data collection process.

Ultimately, the dataset comprised 24,292 participants, including 8,747 males and 15,545 females, with an average age of 20.65 (SD = 2.4) years. Each participant provided complete responses to all four scales, resulting in a collective total of 898,804 item responses.

## Data Records

All files (demographics.csv, phq9.csv, gad7.csv, pss.csv and isi.csv) have been anonymized and both the raw and cleaned data are available in CSV (non-proprietary) formats on the Zenodo platform^36^. All these materials are available at the following link: https://zenodo.org/records/10423537.

In the provided dataset, five distinct CSV files capture the data collected from the research participants. Below, each file’s content and structure list in Table 1.

**Table 1:** The content and structure of dataset files.

File name	context
demographic.csv	**export_id**: A unique identifier for each participant.
	**gender**: Gender of the participant.
	**age**: Age of the participant represented as a number.
	**edu**: Education level of the participant.
	**smoke**: Smoking habits of the participant.
	**drink**: Drinking habits of the participant.
phq9.csv	**export_id**: A unique identifier matching the demographic.csv.
	**score**: The aggregate score of the PHQ9 questionnaire.
	**questionX**: Score for the Xth question.
	**timeX**: Time taken (in seconds) for the participant to answer the Xth question.
gad7.csv	**export_id**: A unique identifier matching the demographic.csv.
	**score**: The aggregate score of the GAD7 questionnaire.
	**questionX**: Score for the Xth question.
	**timeX**: Time taken (in seconds) for the participant to answer the Xth question.
pss.csv	**export_id**: A unique identifier matching the demographic.csv.
	**score**: The aggregate score of the PSS questionnaire.
	**questionX**: Score for the Xth question.
	**timeX**: Time taken (in seconds) for the participant to answer the Xth question.
isi.csv	**export_id**: A unique identifier matching the demographic.csv.
	**score**: The aggregate score of the ISI questionnaire.
	**questionX**: Score for the Xth question.
	**timeX**: Time taken (in seconds) for the participant to answer the Xth question.

## Technical Validation

### Descriptive validation

In the comprehensive psychological screening conducted, data from a total of 24,292 individuals were collected across four distinct scales: PHQ-9, GAD-7, ISI, and PSS. For the PHQ-9 scale, the distribution is as follows: 16,886 individuals (69.51%) reported minimal symptoms, 6,090 (25.07%) displayed mild symptoms, 957 (3.94%) indicated moderate symptoms, 262 (1.08%) had moderately severe symptoms, and 97 (0.40%) exhibited severe symptoms. Within the GAD-7 scale, 20,170 participants (83.03%) had minimal anxiety symptoms, 3,576 (14.72%) reported mild symptoms, 332 (1.37%) had moderate symptoms, 157 (0.65%) showcased severe symptoms, and a minority of 57 individuals (0.23%) had very severe symptoms. For the ISI scale, a significant majority of 22,090 individuals (90.94%) exhibited no clinically significant insomnia, while 1,922 (7.91%) had subthreshold insomnia, 242 (1.00%) had clinical insomnia (moderate severity), and 38 (0.16%) displayed clinical insomnia (severe). Lastly, for the PSS scale, 6,607 respondents (27.20%) showed low stress, 14,884 (61.27%) exhibited moderate stress, 2,687 (11.06%) indicated high perceived stress, and 114 (0.47%) were classified under the very high perceived stress category.

**Fig. 1:**
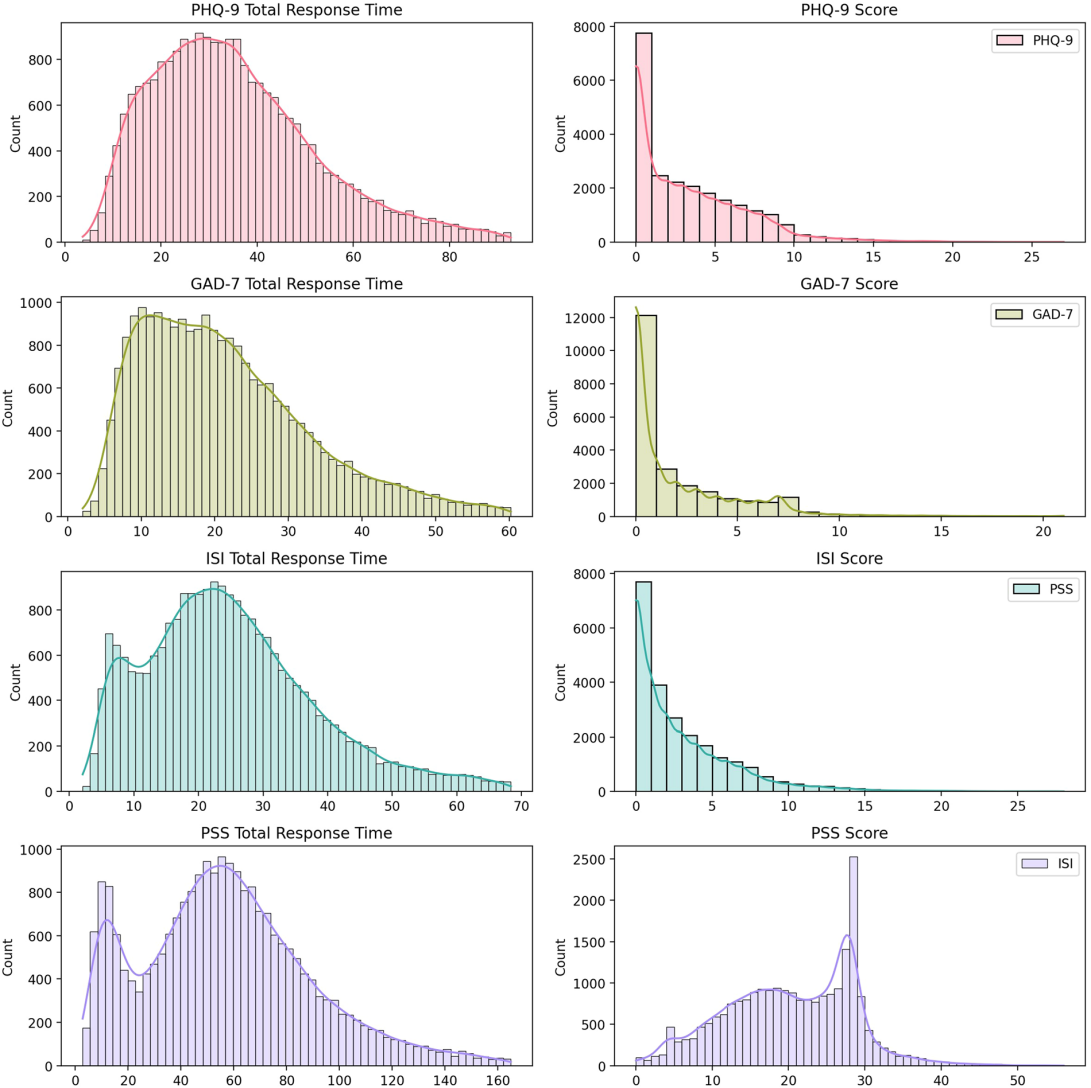
Distributions of total response times and scores for four different psychological assessments: PHQ-9, GAD-7, ISI, and PSS. Each subplot presents a histogram overlaid with a kernel density estimate (KDE) to visualize the distribution of data. (**a,b**) show the total response time and scores for the PHQ-9, respectively; (**c,d**) for the GAD-7; (**e,f**) for the ISI; and (**g,h**) for the PSS. Different colours are used to distinguish between the four assessments. Legends indicate the respective assessment tool in each subplot.

**Fig. 2:**
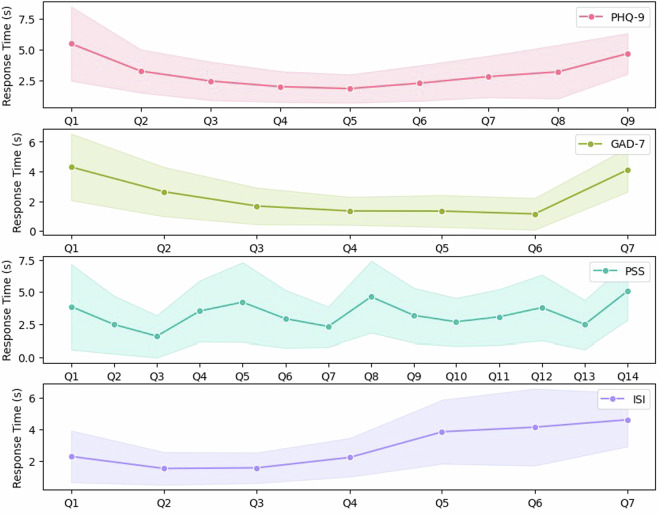
Median response times with error bands for individual questions across four psychological assessment scales: PHQ-9, GAD-7, PSS, and ISI. Each panel presents a line plot of the median response time (in seconds) for each question within the respective scale. The error bands represent standard deviations (scaled by 0.5) around the median estimates.

For the subsequent analyses, we calculated every scale response time’s median and median absolute deviation (MAD) and then excluded participants whose standardized deviation exceeded five times the MAD. Figure 1 illustrates the response time distributions (Fig. 1a,c,e,g) and score distributions (Fig. 1b,d,f,h) for the four scales. It can be observed that the response time distributions for the ISI and PSS scales exhibit distinct bimodal distributions, with clear inflection points at approximately 12 seconds for the ISI and 23 seconds for the PSS. For participants with shorter response times, we tend to consider the presence of careless responding behaviour, and this group should be treated with caution during analysis. Figure 2 shows the response times for individual items on the four scales and the overall variation in response times as the questions progress.

### Quantitative validation

We quantitatively validated our dataset via a word counts effect on response time. To address the issue of non-negative and right-skewed distribution of response time, we performed generalized linear mixed model (GLMM) analyses with word count as an independent continuous variable, and RT as the dependent variable, incorporating subject as random effects. GLMM allows fitting data without the normality assumptions and the need for transformation. It allows differences between individuals to be properly assessed^37^. Analysis was performed using the statsmodels^38^ package in the Python 3.9 environment.

The fixed effect of the intercept was estimated at 2.662 (SE = 0.011, z = 233.506, p < 0.001), indicating the baseline response time. The coefficient for word count was 0.075 (SE = 0.001, z = 117.010, p < 0.001), suggesting a significant positive relationship between word count and response time. Specifically, for each additional word in the count, the response time increased by approximately 0.075 seconds. The random effects included a group variance of 0.849 and a covariance between group intercept and word count of 0.040, with a variance for word count of 0.002. These results indicate that while there is significant between-group variability, the relationship between word count and response time remains robust. The detailed results are shown in Table 2.

**Table 2:** The results from the Generalized Linear Mixed Model (GLMM) analysing the effect of word count on response time, with subject as a random effect.

	Coefficient	Std. Error	z-value	p-value	95% CI
(Intercept)	2.662	0.011	233.506	<0.001	2.640–2.684
Word Count	0.075	0.001	117.010	<0.001	0.074–0.076

## Usage Notes

### Data overview

This dataset provides a comprehensive snapshot of the psychological evaluations conducted between February 27, 2021, and March 17, 2021, at Xinxiang Medical University. It comprises data from 24,292 students who utilized a mobile application to respond to items from key psychological scales. These scales include the PHQ9, GAD7, ISI, and PSS. Alongside the primary responses, we also captured data related to the time participants took to respond, offering an added dimension to the dataset.

### Potential applications

The depth and breadth of this dataset, with its robust sample size of N = 24,292, make it an invaluable asset for numerous research initiatives. The dataset offers opportunities to explore various research questions, from examining the prevalence of specific mental health symptoms among university students to probing correlations between different psychological conditions. Importantly, the data was collected during the COVID-19 pandemic, providing a unique context to investigate the impact of this global crisis on mental health.

Furthermore, the inclusion of response time data adds a novel dimension to traditional psychological evaluations. It allows for an investigation into participants’ engagement during assessments and comprehension of their thought processes. In large-scale psychological screenings, careless responding is likely present in all survey data^6^. Factors such as survey design and incentive methods can contribute to this issue, and response time is an effective means of identifying such behaviour^6^. Additionally, by analysing continuous response time sequences, researchers can model and classify participants’ response behaviours, enabling more precise interpretation of psychological screening results. Furthermore, this analysis can inform the development of more efficient scales and questionnaires, thereby improving the overall accuracy and effectiveness of psychological screenings.

### Limitations

While the dataset presents a robust resource, its utilization comes with several caveats. Firstly, the inherent nature of self-report questionnaires introduces potential biases, such as social desirability or recall bias, where participants might either opt for socially acceptable answers or face challenges in accurately recalling past experiences. Additionally, the data is predominantly derived from students at Xinxiang Medical University, potentially limiting its generalizability to broader populations or students from different cultural and socio-economic backgrounds. Lastly, the inclusion of response time data, though insightful, brings its own challenges. The factors influencing response times can range from genuine contemplation to mere distractions, necessitating that researchers handle this data judiciously, ensuring they account for potential variables in their analyses.

## Supplementary information

Supplement Table Measurement instruments

## Data Availability

All code for formatting, cleaning, and quality assurance was written in Python (python.org) with use of the NumPy (numpy.org) and Pandas (pandas.pydata.org) libraries. Code is available on https://github.com/njnklab/response-time-dataset.
