# Supplementary material for: Temporal dynamics in psychological assessments: a novel dataset with scales and response times
Source: Sci Data. 2024 Sep 27;11:1046. doi: 10.1038/s41597-024-03888-8 (PMC11437125; doi:10.1038/s41597-024-03888-8)
Supplement: Supplementary file 1 — Supplement Table Measurement instruments [file 41597_2024_3888_MOESM1_ESM.docx]

Supplement Table Measurement instruments

|  | content | scoring criteria | item |
| --- | --- | --- | --- |
| Demographics | Age, Gender, Education, Smoke, Drink |  | 5 |
| PHQ-9^28^ | The PHQ-9 is a clinically validated screening tool that is used to identify and assess the severity of depression in individuals. It consists of 9 questions, each scored from 0 to 3, with a total possible score range of 0-27. The scores represent the different levels of depression severity. Cronbach's alpha for the internal consistency reliability of the Chinese version of the PHQ-9 was 0.86 for the entire scale^29^. | minimal (0-4)  mild (5-9)  moderate (10-14)  moderately severe (15-19)  severe (20-27) | 9 |
| GAD-7^30^ | The GAD-7 is a self-reported questionnaire for screening and severity measuring of generalized anxiety disorder (GAD). Like PHQ-9, it contains 7 questions, with each being scored from 0 to 3, and having a total score range of 0-21. The scores interpret the different severity of anxiety. Cronbach's alpha for the Chinese version GAD-7 was 0.84^31^. | minimal (0-4)  mild (5-9)  moderate (10-13)  severe (14-18)  very severe (19-21) | 7 |
| PSS^32^ | The Perceived Stress Scale (PSS) is a widely used psychological instrument for measuring the perception of stress. It is designed to gauge the degree to which situations in one's life are appraised as stressful. The scores on the PSS can range from 0 to 56, with higher scores indicating higher perceived stress. The PSS scores are obtained by reversing the scores on the four positive items, e.g., 0=4, 1=3, 2=2, etc. and then summing across all scale items. The Cronbach's  coefficient for the Chinese version of this scale is 0.83^33^. | low stress (0-14)  moderate stress (15-28)  high perceived stress (29-42)  very high perceived stress (43-56) | 14 |
| ISI^34^ | The Insomnia Severity Index (ISI) is a tool used to measure a person's perceived insomnia severity. It is a brief self-report instrument with 7 items, assessing the severity of sleep-onset and sleep maintenance difficulties, satisfaction with current sleep patterns, interference with daily functioning, noticeability of impairment attributed to the sleep problem, and level of distress or concern caused by the sleep problem. The scores range from 0 to 28, where higher scores represent more severe insomnia symptoms. In a Chinese study, the Cronbach's alpha  coefficient for the total scale of the questionnaire was reported as 0.83^35^. | no clinically significant insomnia (0-7)  subthreshold insomnia (8-14)  moderate severity clinical insomnia (15-21)  severe clinical insomnia (22-28) | 7 |
